# Non-invasive investigation of early kidney damage in streptozotocin-induced diabetic rats by intravoxel incoherent motion diffusion-weighted (IVIM) MRI

**DOI:** 10.1186/s12882-021-02530-8

**Published:** 2021-09-26

**Authors:** You-Zhen Feng, Xiao-Qiao Chen, Zhong-Yuan Cheng, Qi-Ting Lin, Ping-Kang Chen, Ding-Kun Si-Tu, Rui Cao, Long Qian, Baoli Heng, Xiang-Ran Cai

**Affiliations:** 1 Medical Imaging Center, Jinan University First Affiliated Hospital, No.613 West Huangpu Avenue, Tianhe District, Guangzhou, 510630 Guangdong China; 2 Medical Imaging Center, The Eighth Affiliated Hospital of Sun Yat-Sen University, Shenzhen, Guangdong China; 3 Nephrology Department, Jinan University First Affiliated Hospital, Guangzhou, Guangdong China; 4 GE Healthcare, Beijing, China; 5 Department of Biomedical Engineering, Peking University, Beijing, 100871 China; 6 Yingde Base, Institute of Kidney Surgery, Jinan University, Guangzhou, Guangdong China; 7 Department of Urology, Jinan University First Affiliated Hospital, Guangzhou, China

**Keywords:** Early diabetic nephropathy, Noninvasive, Intravoxel incoherent motion diffusion, Pathological correlation, Streptozotocin

## Abstract

**Background:**

The current study investigated the performance of intravoxel incoherent motion diffusion (IVIM) technology in monitoring early renal injury in streptozotocin rats.

**Methods:**

Forty-eight Sprague-Dawley (SD) rats were divided into a control group and a diabetic mellitus (DM) group. Six rats in each group were randomly selected for MR scans at four different time points (0, 4, 8, and 12 weeks). The IVIM-derived parameters (D, D*, *f* and ADC values) of the renal cortex (CO), outer and inner stripe of the outer medulla (OS, IS), and internal medulla (IM) were acquired. Changes in each IVIM-derived parameter over time were analyzed, and differences between the two groups at each point were assessed. The associations between the IVIM parameters and IV collagen expression, urine volume (UV), blood urea nitrogen (BUN), and serum creatinine (Scr) were investigated.

**Results:**

The D and D* values of CO and the ADC values of CO, OS, IS and IM displayed significantly different trends between the two groups over time (*P*<0.05). In addition, significant correlations were discovered between the D* value of CO and UV and BUN (*r*=0.527, *P*=0.033; *r*=0.617, *P*=0.005), between the ADC value of IM and BUN (*r*=0.557, *P*=0.019) and between the *f* value of IM and BUN (*r*=0.527, *P*=0.033). No correlation was found between IVIM parameters and IV collagen expression and Scr.

**Conclusions:**

IVIM is a potential sensitive and noninvasive technology for the simultaneous assessment of early renal cortical and medullary injuries induced by diabetes.

**Supplementary Information:**

The online version contains supplementary material available at 10.1186/s12882-021-02530-8.

## Background

Diabetic nephropathy (DN) is one of the most common systemic microvascular complications of diabetes mellitus (DM) and is considered the leading cause of mortality in patients with DM. However, in the early stage of DN, its clinical diagnosis is not straightforward due to the lack of obvious clinical manifestations of nephropathy. Moreover, traditional blood and urine tests are useless in the early detection of renal dysfunction in patients with DM. Hence, it is urgent to explore an effective method with high sensitivity to identify early kidney injury in DN.

In past decades, DN has long been considered a kind of glomerular disease, and tubular-interstitial damage is regarded as a secondary change [1]. However, in recent years, investigators believe that the impairment of renal tubules precedes the occurrence of glomerular lesions [2–5], and renal tubulointerstitial abnormalities are more likely to be the early modification of DN [5, 6]. Additionally, Vallon et al. [2] indicated that the glomerular filtration rate was determined by the balance of forces between primary tubular and primary vascular events. Taken together, these findings may have a huge influence on the early diagnosis, effective treatment and prognosis of DN.

As a relatively simple and noninvasive tool, previous studies suggested that renal quantitative diffusion weighted imaging (DWI) could potentially play a role in the diagnosis of DN [7–9]. Cakmak et al. [7] revealed that there was a significant correlation between the renal ADC values and clinical stages of DN. Chen et al. [8] speculated that apparent diffusion coefficient (ADC) value may be more sensitive than the urine albuminuria excretion rate in reflecting early-stage kidney injury in DN patients. However, the ADC value based on a single exponential model is approximated as the average diffusion coefficient of various constituent components contained in biological tissue [10], and it does not reflect the true state of dispersion of the renal parenchyma. Le Bihan et al. [11] proposed a two-exponential model of diffusion MRI, named intravoxel incoherent motion (IVIM), to simultaneously estimate tissue diffusivity and microcapillary perfusion. Four parameters, including slow ADC (D, mm^2^/s), fast ADC (D*, mm^2^/s), fraction of fast ADC (*f*, %), and standard ADC (ADC, mm^2^/s), can be calculated according to the IVIM model.

The limitations of the single exponential model become more apparent due to the complexity of the renal structure and function, including vascular flow, tubular flow and passive diffusion [11, 12]. The application of IVIM has been reported in research articles on diabetic renal injury [9, 13–15]. On this basis, our team also conducted a pilot study using IVIM to evaluate the kidneys of patients with early DN [9]. The preliminary results showed that the changes in D, D* and *f* values were more sensitive than the ADC value in detecting early changes in kidneys in diabetic patients before the occurrence of microalbuminuria (MAU). However, this study lacked pathological evidence to further verify our hypothesis because our participants were human beings.

Derived from the aforementioned literature, we hypothesized that IVIM could help us evaluate the early changes in renal functions in pathologically confirmed DM rats. To achieve our goal, rat models of DM were first established, and then the changes in renal function in DM rats were dynamically monitored using IVIM technology at distinct time points.

## Methods

### Animal model

This study was approved by the Animal Experimental Ethical Inspection committee of our university, and the experimental process abided strictly by the animal ethics regulations. Sixty-six male Sprague-Dawley rats weighing between 240 and 250 g were purchased from Jinan Pengyue (Experimental Animal Breeding Co., Ltd., with license number of SCXK (Yue) 20140007). The rats were housed in a well-ventilated environment maintained at 18–22°C, with a relative humidity of 60–70% and 12 h of light per day. All rats had free access to food and water throughout the study.

All rats were randomly divided into 2 groups, the DM and control groups. After fasting for 8 hours, DM group rats were induced by an intraperitoneal injection of streptozocin (STZ; Sigma) at a dose of 55 mg/kg. Control rats received an equal volume of citrate buffer via intraperitoneal injection. Fasting blood glucose (fasting 12 hours) from tail vein samples was measured at 72 hours after STZ injection. Rats with blood glucose levels greater than 16.7 mmol/L after STZ administration were included in the study.

A total of 26 rats were successfully induced by STZ in the DM group; however, two rats were sacrificed caused by anesthesia during the study. Thus, the final 24 rats were included and then randomly subdivided into 4 subgroups (6 rats in each subgroup): 0 w, 4 w, 8 w, and 12 w, which means 0, 4, 8, 12 weeks after induction of diabetes, respectively (Supplemental Figure 1). Another 24 rats in the control group were also randomly allocated to each subgroup as a control. During the study, at each time point (0, 4, 8 and 12 weeks) after induction of diabetes, the rats in the corresponding subgroups were selected for MRI examination. All rats were placed in metabolic cages for collection of 24-h urine samples before MRI. Body weight and blood glucose levels were also measured before MRI. Other clinical indices, including urine creatinine, urea nitrogen, blood urea nitrogen (BUN) and serum creatinine (Scr), were measured using an automatic biochemistry analyzer (Hitachi Model 7600, Japan). These rats were then euthanized via intraperitoneal injection of 0.3% sodium pentobarbital (10 ml/kg), and the kidneys were excised for histopathological examination.

### MR protocol

The anesthetized rats (0.3% sodium pentobarbital, 2 ml/kg intraperitoneally) were scanned using 3T MRI (Discovery MR750, GE Healthcare) with an HD wrist array upper coil. During imaging acquisition, rats were placed in a prone and head advanced position.

MR sequences and parameters were as follows: T2-weighted imaging (T2WI) adopted fast relaxation fast spin echo sequence, repetition time = 3690 ms, echo time = 73 ms, field of view= 7.0 cm×5.6 cm; slice numbers = 7, layer thickness = 1.8 mm, layer interval = 0.2 mm, bandwidth = 15.63 kHz, matrix size = 160×160. IVIM images were acquired in the coronal plane using a spin-echo echo planar imaging sequence, with the following parameters: repetition time = 3500 ms, echo time = 70.4 ms, field of view=8.0 cm×4.8 cm; slice numbers=7, layer thickness = 3.0 mm, layer interval = 0.2 mm, bandwidth = 167 kHz, matrix size = 128×64; and 12 b values (0, 20, 30, 50, 80, 100, 150, 200, 400, 600, 700, 800 s/mm^2^). The total acquisition time was approximately 2 minutes 10 seconds.

### Image postprocessing

IVIM parameter values were calculated using the following equation [10]:

1 $${S}_{\mathrm{b}}/{S}_0=f\exp \left(- bD\ast \right)+\left(1-f\right)\exp \left(- bD\right)$$

where *S*_0_ and *S*_b_ are the signal intensities without and at a given *b* value, respectively. D is the true water molecule diffusion coefficient; D* is the perfusion coherence diffusion coefficient, i.e., pseudodispersion, which can reflect changes in blood perfusion [16]. D* refers to the irregular movement of the liquid in the irregular lumen. The average capillary length, blood flow velocity and blood vessel shape can affect the D* value. D* may also be affected by renal tubules, mean length of collection tubes, and fluid flow rate in kidney studies. *f* is the perfusion-related volume fraction, representing the volume ratio of the diffusion caused by the microcirculation perfusion effect in the overall diffusion effect of the voxel. The larger the *f* value is, the denser the capillary distribution is. In the kidney, the *f* value is not only related to blood vessels but also affected by renal tubules and collecting ducts. ADC is closely related to the b value and is not accurate and objective.

**Fig. 1:**
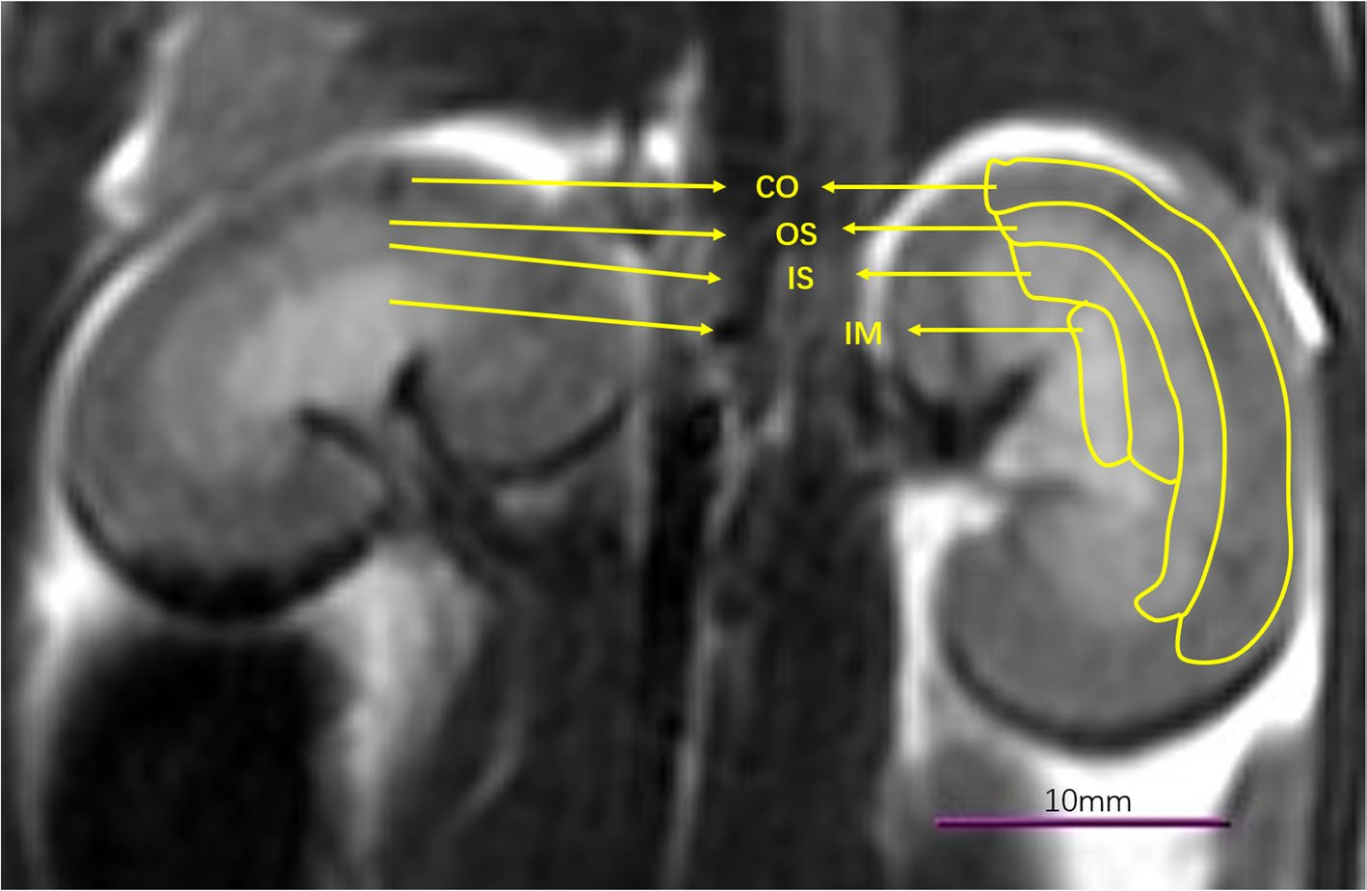
Placement of regions of interest (ROI) into the *b*_0_ image. CO, cortex; OS, outer stripe of the outer medulla; IS, inner stripe of the outer medulla; and IM, inner medullary

**Fig. 2:**
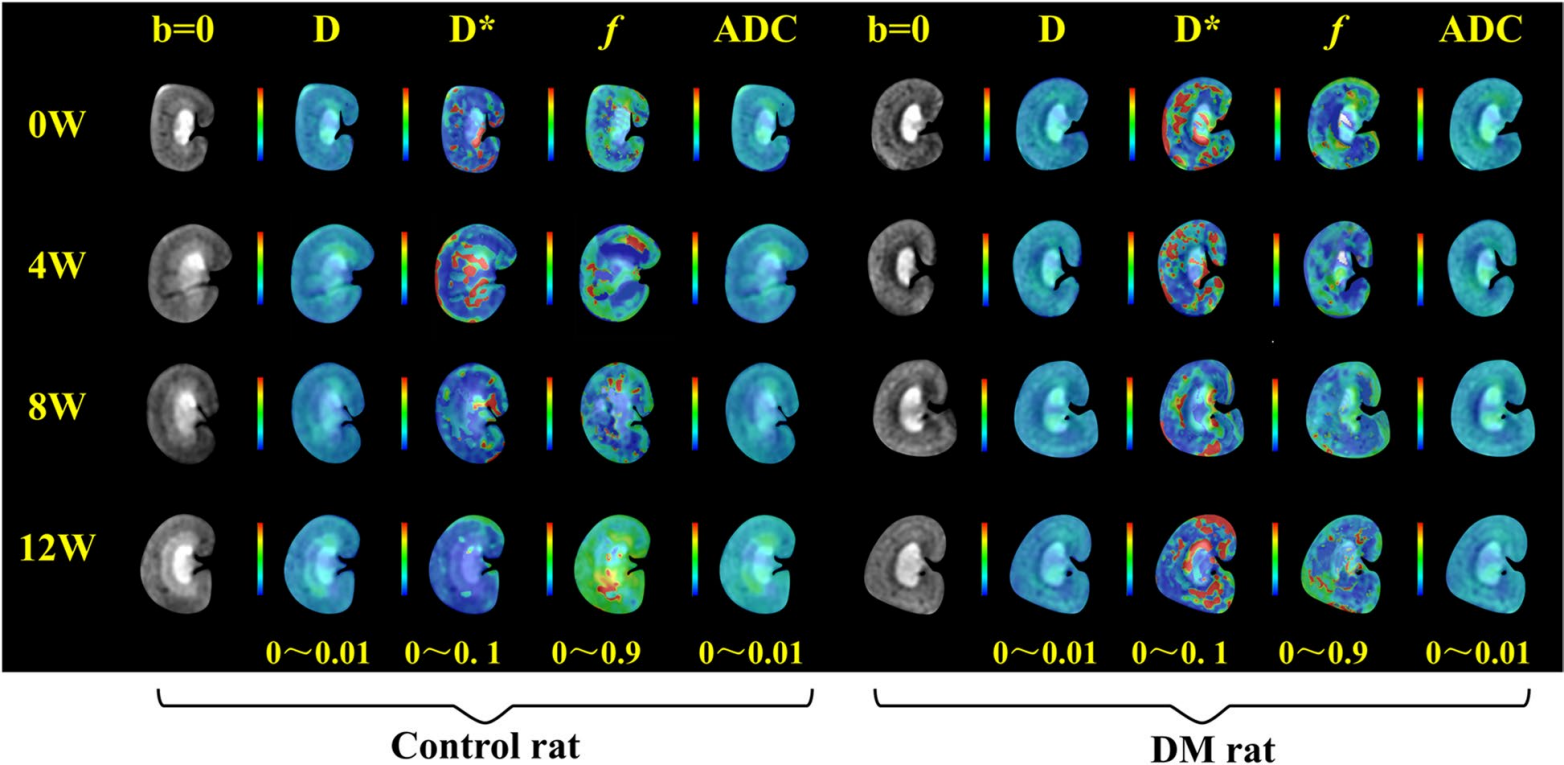
IVIM parametric maps are shown for rats at 0, 4, 8, and 12 weeks after streptozotocin-induced diabetes (DM Group) and citrate buffer injection (Control Group)

The IVIM sequence image raw data were transmitted to the functool software and MADC postprocessing software of GE's ADW4.5 workstation for image postprocessing and analysis, and the IVIM parametric images were obtained. The values of IVIM parameters were independently measured by two radiologists with 6 years and 23 years of diagnostic imaging experience. Regions of interest (ROIs) were drawn manually over the right renal cortex (CO), outer stripe of the outer medulla (OS), inner stripe of the outer medulla (IS) and internal medulla (IM), avoiding the renal sinus and blood vessels on the DWI (b=0) images, and transferred to the various IVIM parametric maps (Fig. 1). The IVIM parametric mapping is shown on Fig. 2. Three continuous coronal images at the level of the renal hilum were used for quantification to optimize the signal-to-noise ratio, which refers to previous research [17, 18]. The ROI sizes of each CO, OS IS and IM are approximately 37-43 mm^2^, 27-33 mm^2^, 17-23 mm^2^ and 7-10 mm^2^, respectively.

### Pathological analysis

A total of 24 rats in the DM groups were sacrificed with an overdose of anesthesia with 0.3% sodium pentobarbital (10 ml/kg). The weights of the rat kidneys were subsequently measured. The kidneys were then fixed in 10% neutral formalin solution for histological examination. Tissues were embedded in paraffin wax, cut into 3-μm sections and stained with hematoxylin and eosin (H&E). Sections of 3-μm thickness were stained with Sirius Red for collagen quantification, which could be considered a marker of fibrosis. Sections were then analyzed and photographed by a pathologist (B.D., 20 years of pathology experience) who was blind to the MR findings.

### Statistical analysis

Statistical analysis was performed using SPSS v. 20.0 software (Chicago, IL). All results are expressed as the mean ± standard deviation (M ± SD), and *P* < 0.05 was considered to be statistically significant. The values of body weight, kidney weight ratio, urine volume (UV), BUN, Scr and MRI indices in the renal cortex of the rats were compared using independent two-sample *t*-tests at each time point. To estimate the time and group differences as well as their mixed interactions of the IVIM-derived indices, repeated-measures ANOVA was applied in the current study. Post hoc independent two-sample *t*-tests were further employed to compare the group differences at distinct time points. The associations between the IVIM-derived parameters and histopathological or biochemical values were quantified using Pearson and Spearman correlation analysis.

## Results

### Animal and laboratory parameters

After 72 hours of STZ injection, the typical characteristics of the diabetic rats developed gradually, including polyphagia, polydipsia, polyuria, weight loss, apathy and poor fur color. Cataracts were discovered at the 7^th^ week. In contrast, the rats in the control group displayed a good mental state, a highly sensitive response, and gradually increased body weight and free movement. The body weights of diabetic rats continued to decline over time. The body weights were significantly lower than the body weights of rats in the control group from the 4^th^ to 12^th^ weeks. In contrast, the ratios of kidney weight/body weight in diabetic rats tended to increase after the 4^th^ week (*P* < 0.05). The UV values of diabetic rats increased significantly at the 4^th^ and 8^th^ weeks and decreased at the 12^th^ week compared with the control group (*P* < 0.05). Both the serum BUN and Scr values in diabetic rats had an increasing trend over time. Intergroup differences were discovered in serum BUN from the 4^th^ to 12^th^ weeks (*P* < 0.05), but no significant differences were found in serum Scr at any time point between the two groups (Table 1).

**Table 1:** Comparison of body weight, kidney weight/body weight, urine volume, BUN and Scr values between the two groups

		Body weight(g)	Kidney weight/body weight (‰)	Amount of urine(ml)	BUN (mmol/L)	Scr (μmol/L)
0 w	Control (*n*=6)	338.64±15.816	7.71±0.646	39.87±8.648	5.79±0.587	23.17±4.446
	DM (*n*=6)	334.72±16.531	8.29±0.634	42.15±8.701	6.25±0.447	24.33±3.777
4 w	Control (*n*=6)	427.78±19.262	6.67±0.829	43.98±22.537	5.89±0.981	24.00±2.000
	DM (*n*=6)	339.42±41.721*	10.28±0.641*	188.78±44.316*	13.51±2.399*	27.33±4.502
8 w	Control (*n*=6)	489.59±25.149	5.81±0.351	48.63±30.142	5.96±1.032	24.22±3.251
	DM (*n*=6)	327.60±31.998*	10.88±1.047*	271.07±109.045*	13.22±2.371*	27.33±3.724
12 w	Control (*n*=6)	522.40±10.944	5.89±0.304	39.57±25.146	6.01±1.022	25.36±4.269
	DM (*n*=6)	301.39±47.567*	13.13±2.530*	126.22±29.715*	14.19±3.295*	30.17±7.512

Note: * the results of *t*-test for two groups of two samples at each time point (*P* < 0.05)

### Comparisons of IVIM parameters

**Fig. 3:**
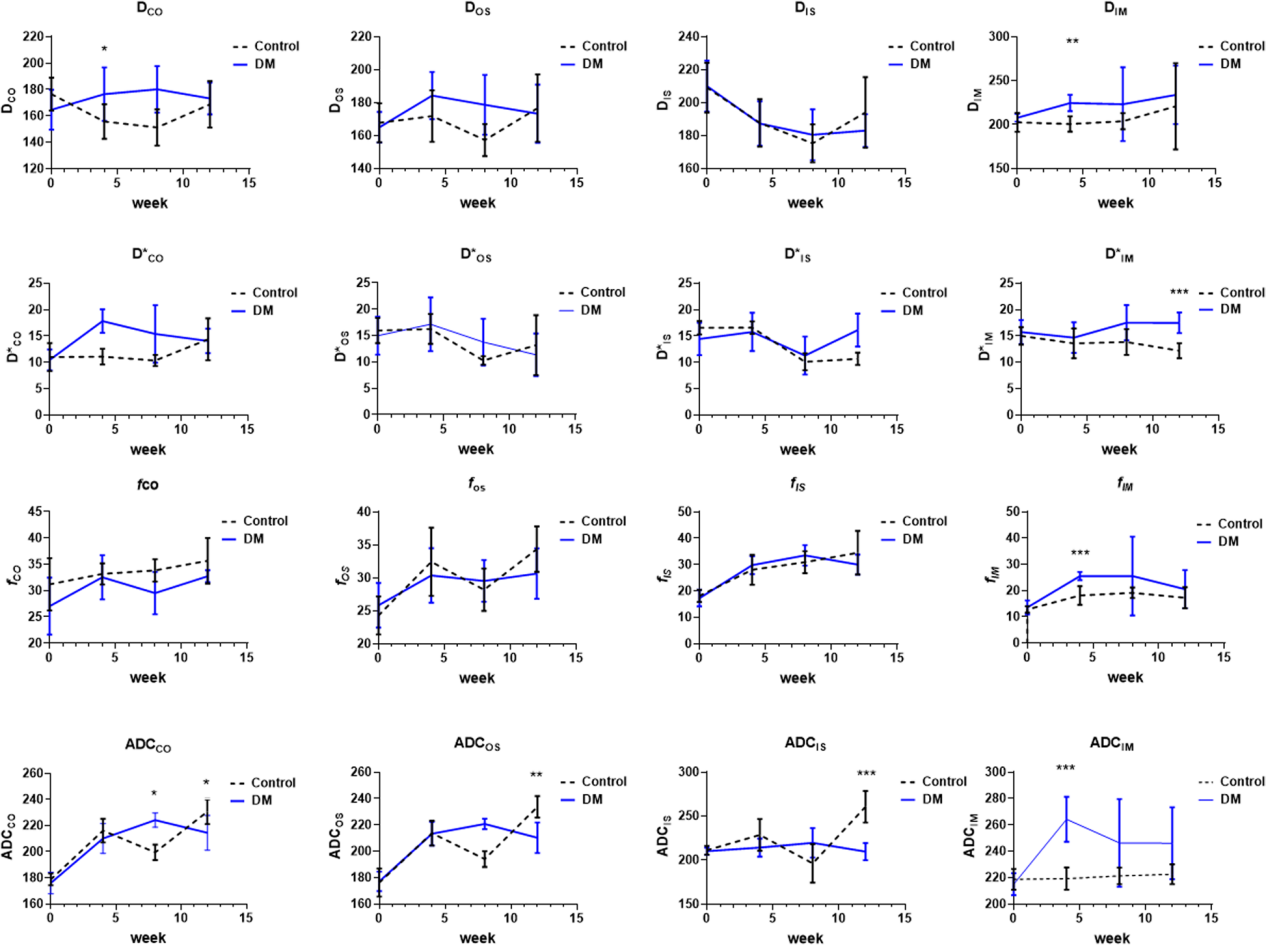
The ADC, D, D*, and *f* values of CO, OS, IS, and IM in the DM and control groups at each time point (0, 4, 8, and 12 weeks). CO, cortex; OS, outer stripe of the outer medulla; IS, inner stripe of the outer medulla; and IM, inner medullary. ADC (standard ADC, mm^2^/s), apparent diffusion coefficient; D (slow ADC, mm^2^/s), extravascular effects of passive diffusion; D* (fast ADC, mm^2^/s), pseudodiffusion coefficient; *f,* (fraction of fast ADC, %), perfusion fraction

Twelve out of 16 IVIM parameters in the kidneys of diabetic rats demonstrated alterations over time, except for D_CO_, D_OS_, D_IM_ and D*_IM_ (P_1_ values shown in Table 2 and Fig. 3). A total of 6 parameters displayed a significant difference in the difference of the IVIM parametric means between the two groups over time, including D_CO_, D*_CO_, ADC_CO_, ADCos, ADC_IS_ and ADC_IM_ (*P*<0.05, as P_2_ values exhibited in Table 2 and Fig. 3). In the DM group, most IVIM parameters showed a trend of rising first and then falling over time. However, the *f* values of the renal cortex and medulla slowly increased, and the D_IS_, D_IM_ and D*_IM_ values had a declining trend over time. In contrast, none of the parameters in the controls displayed similar trends.

**Table 2:** The change trend comparison of IVIM parameters between rat groups over time

		Time		Time*Group		Intersubjective	
		F_**1**_	***P***_***1***_	F_**2**_	***P***_***2***_	F_**3**_	***P***_***3***_
**CO**	**D**	0.34	0.799	3.38	**0.031**	13.45	**0.004**
	D*	4.09	**0.015**	3.50	**0.028**	30.49	**<0.001**
	***f***	4.58	**0.009**	0.71	0.556	5.04	**0.049**
	**ADC**	54.37	**<0.001**	10.66	**<0.001**	0.00	0.974
**OS**	**D**	1.50	0.244	1.75	0.192	0.00	0.093
	D*	7.47	**0.001**	1.92	0.147	0.05	0.822
	***f***	12.19	**<0.001**	0.94	0.433	2.38	0.154
	**ADC**	52.86	**<0.001**	14.20	**<0.001**	0.29	0.604
**IS**	**D**	8.97	**0.002**	0.60	0.562	0.11	0.743
	D*	11.76	**<0.001**	5.07	1.006	1.20	0.300
	***f***	21.96	**<0.001**	1.17	0.337	0.08	0.782
	**ADC**	6.98	**0.002**	11.02	**<0.001**	34.49	**<0.001**
**IM**	**D**	1.37	0.280	0.26	0.738	4.81	0.053
	D*	0.76	0.530	1.98	0.139	35.37	**<0.001**
	***f***	5.22	**0.005**	0.67	0.580	5.94	**0.035**
	**ADC**	4.05	**0.016**	3.67	**0.023**	25.40	**0.001**

*Note*: D true diffusivity, D* pseudodiffusion coefficient, *f* perfusion fraction, ADC apparent diffusion coefficient. Data are presented as the mean ± standard deviation. The *P* values in bold type are of statistical significance. D, ADCs are presented in × 10^−5^ mm^2^/s, D* is presented in × 10^−3^ mm^2^/s, and *f* is presented as a percentage. CO, renal cortex, OS, outer stripe of the outer medulla, IS, inner stripe of the outer medulla, IM, internal medulla

P_1_ indicates a difference in the changes in IVIM parameters over time between the two groups

P_2_ indicates a difference in the difference of the IVIM parametric means between the two groups over time

P_3_ indicates a difference in the IVIM parametric means between the two groups

At 4 weeks, the D_CO_, D_OS_, D_IM_, D*_CO_, D*_IM_, *f*_IM_ and ADC_IM_ values of diabetic rats were greater than the comparable values of the controls, and significant differences were discovered in the D*_CO_, D_IM_, *f*_IM_, and ADC_IM_ values between the two groups (*P* < 0.05) (Table 3 and Fig. 3). The D and *f* values of CO and the D* value of IM did not change significantly in diabetic rats until the 8^th^ week. The ADC values of IS showed a significant difference between the two groups at the 12^th^ week (Table 3 and Fig. 3).

**Table 3:** IVIM parameters between rat groups at different time

			0 week	4 week	8 week	12 week
**CO**	**D**	**Control**	176.55±12.42	155.83±13.12	151.33±9.79	176.67±20.50
		**DM**	164.73±15.14	176.50±20.25	1778.67±18.15	173.33±17.70
		**t/*P***	0.43/0.530	9.07/**0.013**	1.31/0.279	0.49/0.501
	D*	**Control**	11.01±2.66	11.70±1.49	10.37±1.06	12.40±1.24
		**DM**	10.48±1.99	17.81±2.23	15.40±5.46	14.07±2.31
		**t/*P***	1.02/0.336	1.07/0.325	1.70/0.222	2.65/0.135
	***f***	**Control**	31.20±4.99	33.18±2.00	33.85±2.14	35.67±4.33
		**DM**	27.05±5.40	2.53±4.18	29.53±4.02	32.73±1.13
		**t/*P***	0.67/0.434	0.06/0.817	0.04/0.850	0.10/0.762
	**ADC**	**Control**	178.79±4.48	216.17±9.07	199.50±5.96	230.83±9.52
		**DM**	176.00±8.32	210.17±11.43	224.33±5.47	214.50±13.40
		**t/*P***	1.39/0.266	2.13/0.175	8.98/**0.013**	5.39/**0.043**
**OS**	**D**	**Control**	167.76±11.81	171.83±15.57	157.33±9.79	176.67±20.51
		**DM**	165.00±9.27	184.33±14.36	178.67±18.15	173.33±17.7
		**t/*P***	0.27/0.613	3.34/0.098	2.55/0.142	0.13/0.730
	D*	**Control**	15.96±2.39	16.27±2.84	10.28±0.81	13.20±5.68
		**DM**	14.98±3.60	17.15±5.10	13.78±4.41	11.37±4.04
		**t/*P***	0.02/0.896	1.49/0.251	0.82/0.388	1.54/0.243
	***f***	**Control**	24.85±3.00	32.43±5.17	28.20±3.22	34.35±3.46
		**DM**	23.15±2.83	30.35±4.16	29.53±3.17	30.65±3.84
		**t/*P***	0.16/0.698	0.12/0.738	1.69/0.233	0.08/0.790
	**ADC**	**Control**	176.09±10.69	213.67±9.48	194.00±6.00	233.67±8.82
		**DM**	177.00±7.46	213.33±9.03	220.67±3.98	210.17±11.62
		**t/*P***	0.02/0.899	3.33/0.098	1.62/0.231	23.02/**0.001**
**IS**	**D**	**Control**	209.11±15.09	187.67±14.46	175.33±11.55	194.00±21.43
		**DM**	210.0±15.58	187.33±13.52	180.50±15.50	183.00±9.96
		**t/*P***	0.60/0.475	0.02/0.891	0.78/0.398	0.65/0.438
	D*	**Control**	16.57±1.27	16.60±1.19	10.12±1.61	10.86±1.16
		**DM**	14.44±3.07	15.78±3.62	11.30±3.61	16.13±3.14
		**t/*P***	2.16/0.172	0.10/0.754	0.03/0.864	2.73/0.129
	***f***	**Control**	18.13±2.35	28.05±5.68	30.93±4.18	34.53±8.28
		**DM**	17.28±3.12	29.82±3.42	33.52±3.86	30.03±3.79
		**t/*P***	0.71/0.419	0.03/0.858	1.19/0.301	0.03/0.869
	**ADC**	**Control**	211.13±5.06	228.50±18.09	196.17±21.87	260.50±17.90
		**DM**	210.00±4.15	214.17±10.21	219.50±16.81	209.50±9.85
		**t/*P***	0.06/0.812	0.68/0.429	0.00/1.000	26.81/**<0.001**
**IM**	**D**	**Control**	202.50±10.86	200.67±8.73	203.83±9.09	220.83±49.14
		**DM**	207.83±4.54	224.50±9.33	223.17±41.97	233.83±33.23
		**t/*P***	1.23/0.290	20.86/**0.001**	1.22/0.300	0.29/0.600
	D*	**Control**	15.02±1.68	13.60±2.84	13.84±2.44	12.20±1.44
		**DM**	15.73±2.67	14.68±2.90	17.50±3.37	17.48±1.96
		**t/*P***	0.39/0.550	0.43/0.530	4.63/0.057	28.23/**<0.001**
	***f***	**Control**	12.80±1.20	18.15±3.54	19.13±2.03	17.26±4.02
		**DM**	13.60±2.65	25.50±1.58	25.51±15.02	20.53±7.26
		**t/*P***	0.45/0.520	21.59/**0.001**	1.06/0.330	0.93/0.360
	**ADC**	**Control**	218.67±7.87	219.17±8.45	221.33±6.25	222.50±7.58
		**DM**	215.00±8.37	264.17±17.03	246.33±33.27	246.00±27.26
		**t/*P***	0.61/0.450	33.61/**<0.001**	3.27/0.100	4.14/0.070

*Note*: D true diffusivity, D* pseudodiffusion coefficient, *f* perfusion fraction, ADC apparent diffusion coefficient. Data are presented as the mean ± standard deviation. The *P* values in bold type are of statistical significance. D, ADCs are presented in × 10^−5^ mm^2^/s, D* is presented in × 10^−3^ mm^2^/s, and *f* is presented as a percentage. *CO* renal cortex, *OS* outer stripe of the outer medulla, *IS* inner stripe of the outer medulla, *IM* internal medulla

### Pathological results

#### HE stain

**Fig. 4:**
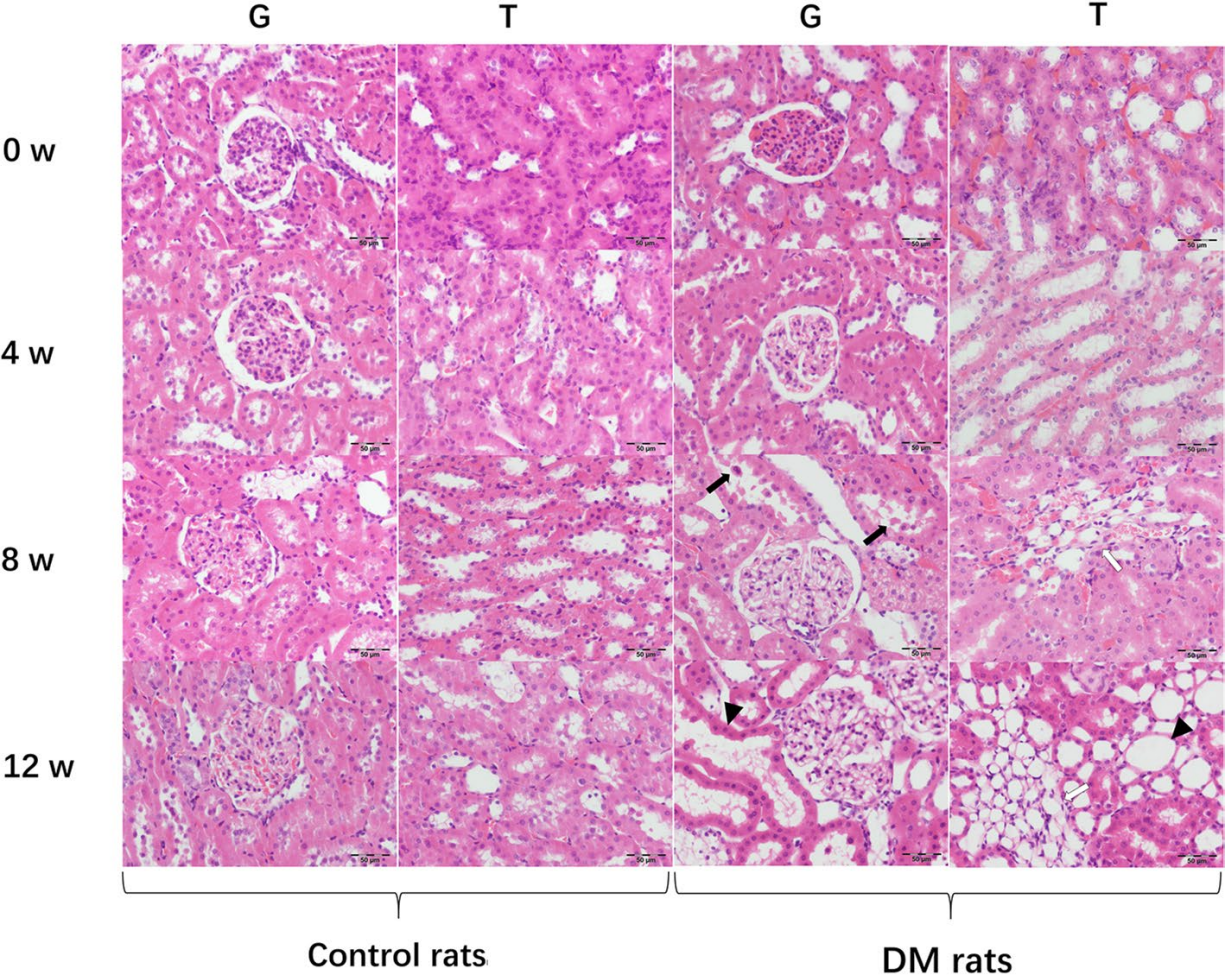
Histological analysis of kidney tissue sections in diabetic rats and controls. Histology of Glomeruli (G) and Renal Tubule (T). All sections were stained with H&E stain. Dilated renal tubules (arrowheads), capillary proliferation (white arrows), renal tubular epithelial necrosis and exfoliation (black arrows) are demonstrated

**Fig. 5:**
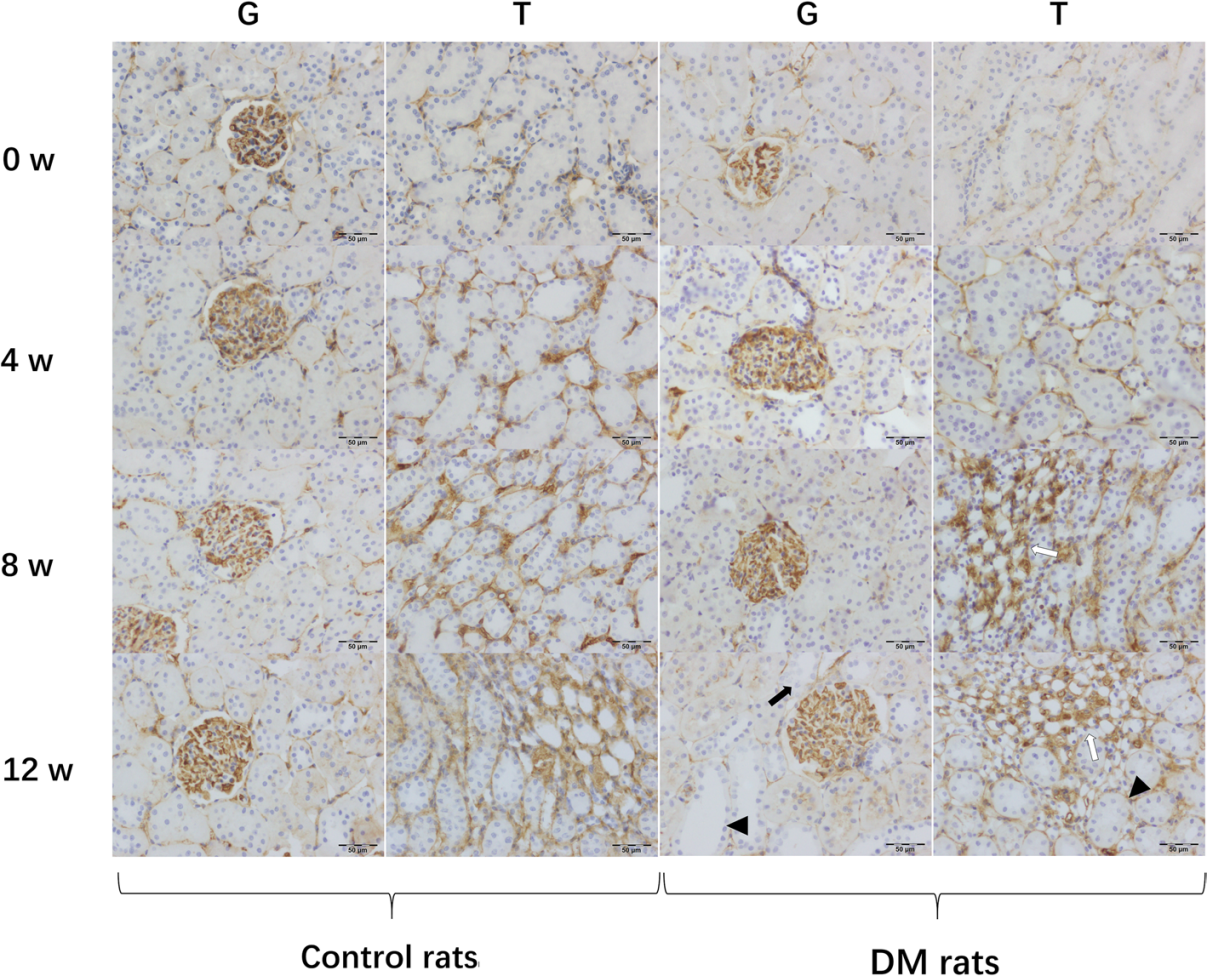
Histological analysis of kidney tissue sections in diabetic rats and controls. Histology of glomeruli (G) and renal tubule (T). All sections were stained with podocin stains. Dilated renal tubules (arrowheads), capillary proliferation (white arrows), renal tubular epithelial necrosis and exfoliation (black arrows) are demonstrated

At the 4^th^ week, the renal glomerular volume and extracellular matrix (ECM) increased slightly in diabetic rats. Furthermore, mild mesangial hyperplasia, vacuolated renal tubular epithelial cells, slightly dilated renal tubules and capillary lumen were also observed. Renal tubular abnormalities in the medulla were more prominent in our results (Figs. 4 and 5).

At the 8^th^ week, swollen glomerular endothelial cells, moderately increased mesangium and ECM, glomerular wall adhesion, moderate capillary lumen expansion, partial renal tubular epithelial necrosis and exfoliation and interstitial endothelial cell infiltration were demonstrated in diabetic rats (Figs. 4 and 5).

At the 12^th^ week, thickened basement membranes of glomeruli and renal tubules were discovered. Severe mesangial and ECM hyperplasia, uneven expansion of the glomerular balloon, adhesion of balloon wall and highly dilated capillary lumen were also shown. Furthermore, tubular atrophy, dilated tubules, necrotic and detached tubular epithelial cells and a large amount of interstitial inflammatory cell infiltration were observed (Figs. 4 and 5).

For the controls, the renal glomeruli and tubules were normal over time. There was no inflammatory cell infiltration or dilated and congested blood vessels in the renal interstitium (Figs. 4 and 5).

#### Collagen IV immunohistochemistry

As shown in the Supplemental Table, the expression of Collagen IV gradually increased in the kidneys of diabetic rats over time. Capillary hyperplasia in the tubulointerstitium gradually became apparent.

### Correlation analysis

The statistical results are shown in Table 4. D*_CO_, ADC_IM_ and *f*_IM_ showed significant positive correlations with UV (D*_CO_: *r*=0.527, *P*=0.033) and BUN (D*_CO_: *r*=0.617, *P*=0.005; ADC_IM_: *r*=0.557, *P*=0.019; *f*_IM_: *r*=0.527, *P*=0.033).

**Table 4:** Correlation analysis between IVIM parameters and IV collagen expression, Urine volume, BUN, and Scr

	IV collagen		Urine volume		BUN		Scr	
	r	***P***	r	***P***	r	***P***	r	***P***
**D**_**CO**_	0.146	0.496	0.34	0.104	0.228	0.284	0.046	0.832
D*_CO_	0.115	0.592	**0.527**	**0.033**^**£**^	**0.617**	**0.005**^**£**^	0.311	0.14
***f***_**co**_	0.347	0.097	0.276	0.192	0.339	0.105	0.313	0.136
**ADC**_**IS**_	-0.202	0.344	0.275	0.193	0.108	0.617	0.061	0.778
**ADC**_**IM**_	0.282	0.182	0.329	0.116	**0.557**	**0.019**^**£**^	0.054	0.801
D*_IM_	0.19	0.373	0.293	0.165	0.163	0.445	0.248	0.242
***f***_**IM**_	0.232	0.275	0.132	0.539	**0.527**	**0.033**^**£**^	-0.055	0.799

*Note*: D true diffusivity, D* pseudodiffusion coefficient, *f* perfusion fraction, ADC apparent diffusion coefficient. The *P* values in bold type are of statistical significance. D, ADC are presented in × 10^−5^ mm^2^/s, D* is presented in × 10^−3^ mm^2^/s, and *f* is presented as a percentage. *CO* renal cortex, *OS* outer stripe of the outer medulla, *IS* inner stripe of the outer medulla, *IM* internal medulla. ^**£**^: *P* value corrected by Bonferroni

## Discussion

In the current study, we used an IVIM model to evaluate the functional fluctuations of kidneys over time in STZ diabetic rats, and correlations were detected between IVIM-derived parameters and histopathological or biochemical indices. Our results demonstrated three important findings. First, 12 out of 16 renal IVIM parameters displayed significant trends over time in our study. In addition, some IVIM parameters, including D_CO_, D*_CO_, ADC_CO_, ADCos, ADC_IS_ and ADC_IM_, revealed significant changes between the two groups. These results suggested a high sensitivity of IVIM in detecting early renal damage in diabetic rats. Second, there were significant correlations between the cortical D* value and UV or BUN, between the f_IM_ and BUN and between the ADC_IM_ and UV, indicating damage to both renal glomeruli and tubules in the early stage of diabetes. Finally, the perfusion (D* and *f* values) and molecular diffusion (D and ADC value) parameters may be sensitive indicators for the detection of cortical and medullary damage in diabetic rats.

Our study found significantly higher Dco, D_IM_ and ADC_IM_ values at the 4^th^ week as well as ADCco at the 8^th^ week in diabetic rats, indicative of an increase in water molecule diffusion of CO and IM. The histological results revealed mildly increased renal glomerular volume and vacuolated renal tubular epithelial cells in the inner medulla, which could contribute to expanding the extracellular space and increasing the diameter of the renal tubule, subsequently resulting in higher D and ADC values. Additionally, previous studies showed doubled tubular cell and luminal diameters, increased cell height and a 37% increase in proximal tubule length at the 7^th^ week, which reflected hyperplasia and hypertrophy of the kidney [19, 20]. In addition, Sigmund et al. [16] demonstrated that reabsorption played a strong role in the increased D value because of the stronger urine output sensitivity of hydration after furosemide administration. This may be another factor for the higher D_IM_ and ADC_IM_ values in diabetic rats. The increased ADC value is at least in part the result of an increase in microcirculation due to hyperfiltration and hyperperfusion [12, 16].

Although the kidney has a certain anti-injury and hypoxia reserve capacity [21–23], the obvious accumulation of ECM, thickened glomerular and tubular basement membrane, mesangial hyperplasia and interstitial inflammatory cell infiltration become more prominent after the 4^th^ week with the further development and deterioration of renal function. These changes will contribute to the increased cell density or decreased extracellular space and consequently the restricted diffusion of water molecules [24]. Therefore, the D and ADC values in our study showed a downward trend from the 8^th^ to 12^th^ weeks and observably lower ADCco and ADCos at the 12^th^ week in diabetic rats. Additionally, the decreased D and ADC values could be related to swollen renal tubular epithelial cells and decreased tubular flow and capillaries [25, 26].

Sigmund et al. [16] indicated that the *f* value is the ratio of the sum of the amount of liquid contained in the renal tubules and capillaries to the total amount of fluid contained in the kidney. A higher *f* value suggested a denser distribution of capillaries, and thus, the sum of lumen diameters was larger [27, 28]. A significantly higher *f*_IM_ at the 4^th^ week in diabetic rats was demonstrated in this study. Additionally, the UV of the diabetic rats was 5-fold greater than the UV of the controls, indicating renal hyperfiltration. At the early stage of diabetic nephropathy, the kidney is in a state of hyperfiltration and hyperperfusion accompanied by an increase in renal fluid load, especially in areas with dense renal tubules. Hence, the increased *f*_IM_ was a good reflection of renal hyperfiltration and hyperperfusion in the early stage of DN. Noticeably, the D* value showed an upward trend of change at the 4^th^ week, especially the D*co value which may indicate that increased perfusion of the kidney occurred as well.

Interestingly, similar to diffusion parameters, a declining trend was also observed in perfusion parameters from the 8^th^ to 12^th^ weeks. The decreased D* and *f* values might be related to the reduced flow rate due to the swelling of tubular epithelial cells and expansion of the lumen in CO and IM [12, 16]. Moreover, numerous studies have suggested that this decrease may be associated with hypoxia [12, 24, 29]. With the progression of the disease, mesangial hyperplasia compresses the surrounding glomerular capillaries, leading to a decrease in renal perfusion. Subsequently, the reduced flow of renal tubules and collecting ducts was followed by the decreased fluid load of the kidneys, which could be another explanation of the reduced perfusion parameter.Furthermore, our results revealed notably increased UV radiation in diabetic rats from the 4^th^ week. Coincidently, D*co was remarkably positively correlated with UV. These findings suggest hyperfiltration and hyperperfusion of the renal cortex. From the onset of diabetes, several vascular and tubular factors contribute to a net reduction in afferent arteriolar resistance, thereby increasing renal blood flow [30, 31]. Various cytokines and growth factors lead to an enlarged nephron size and filtration surface area per glomerulus in response to hyperglycemia [32] particularly hypertrophy of the proximal tubule [33, 34]. Correspondingly, the plasma of the renal cortex will be augmented. Meanwhile, obviously higher D_co_, D_IM_ and ADC_IM_ values were observed at the 4^th^ week in diabetic rats, indicating that the diffusion parameter was sensitive for displaying early changes in the renal cortex and IM. In addition, out of the serum biochemical indicators, only BUN demonstrated a significant increase in the early stage, indirectly verifying that IVIM parameters could be more sensitive than biochemical indices in the detection of pathological changes in early DN.

This study had some limitations. First, STZ was applied to induce diabetes, which can directly destroy the islet cell function of rats. Thus, this diabetic rat model may be more appropriate for type I diabetes. Second, bowel gas and breathing patterns could cause artifacts in imaging, which will affect the accuracy of measurement. In our study, the scan time was shortened as much as possible using the optimized imaging parameters. Additionally, the prone position was applied to reduce breathing artifacts. Moreover, MRI scans were performed during the nighttime, which is helpful for reducing intestinal peristalsis and intestinal gas. Third, the interval between the two adjacent time points was relatively longer. More time points should be considered in future research. Finally, the sample size was relatively small. The sampling error could be reduced by increasing the sample size in the future. Finally, the hyperfiltration may be an important factor that affects IVIM parameters, thus the absence of a direct evidence of hyperfiltration in DM rats is a limitation for this current study.

## Conclusions

In conclusion, our results indicated that IVIM is a potential sensitive and noninvasive technology for the simultaneous assessment of renal cortical and medullary injuries in early DN. Importantly, the current research revealed renal cortex and medulla perfusion abnormalities and molecular diffusion changes in the early stage of diabetes in rats.

## Supplementary Information

**Additional file 1: Supplemental Figure 1**. Experimental protocol and allocations of rats to the two study groups. Six rats in each group underwent (1) streptozotocin or citrate buffer intraperitoneal injection, (2) Blood and urine tests, (3) MR scan (at 0, 4, 8, and 12 weeks after DM induction), (4) Blood and tests, followed by (5) histopathological examination.

**Additional file 2: Supplemental Table.** Expression of Collagen IV in renal tissue of the two groups of rats

## Data Availability

The datasets used and/or analyzed during the current study available from the corresponding author on reasonable request.

## Abbreviations

DN: Diabetic nephropathy
DM: Diabetes mellitus
MAU: Microalbuminuria
IVIM: Intravoxel Incoherent Motion
D: Slow ADC
D*: Fast ADC
*f*: Fraction of fast ADC
CO: Renal cortex
OS: Outer stripe of the outer medulla
IS: Inner stripe of the outer medulla
IM: Internal medulla
UV: Urine volume
BUN: Blood urea nitrogen
Scr: Serum creatinine
ROIs: Regions of interests
STZ: Streptozocin
