# Supplementary figures and images for: Non-invasive investigation of early kidney damage in streptozotocin-induced diabetic rats by intravoxel incoherent motion diffusion-weighted (IVIM) MRI
Source: BMC Nephrol. 2021 Sep 26;22:321. doi: 10.1186/s12882-021-02530-8 (PMC8474753; doi:10.1186/s12882-021-02530-8)

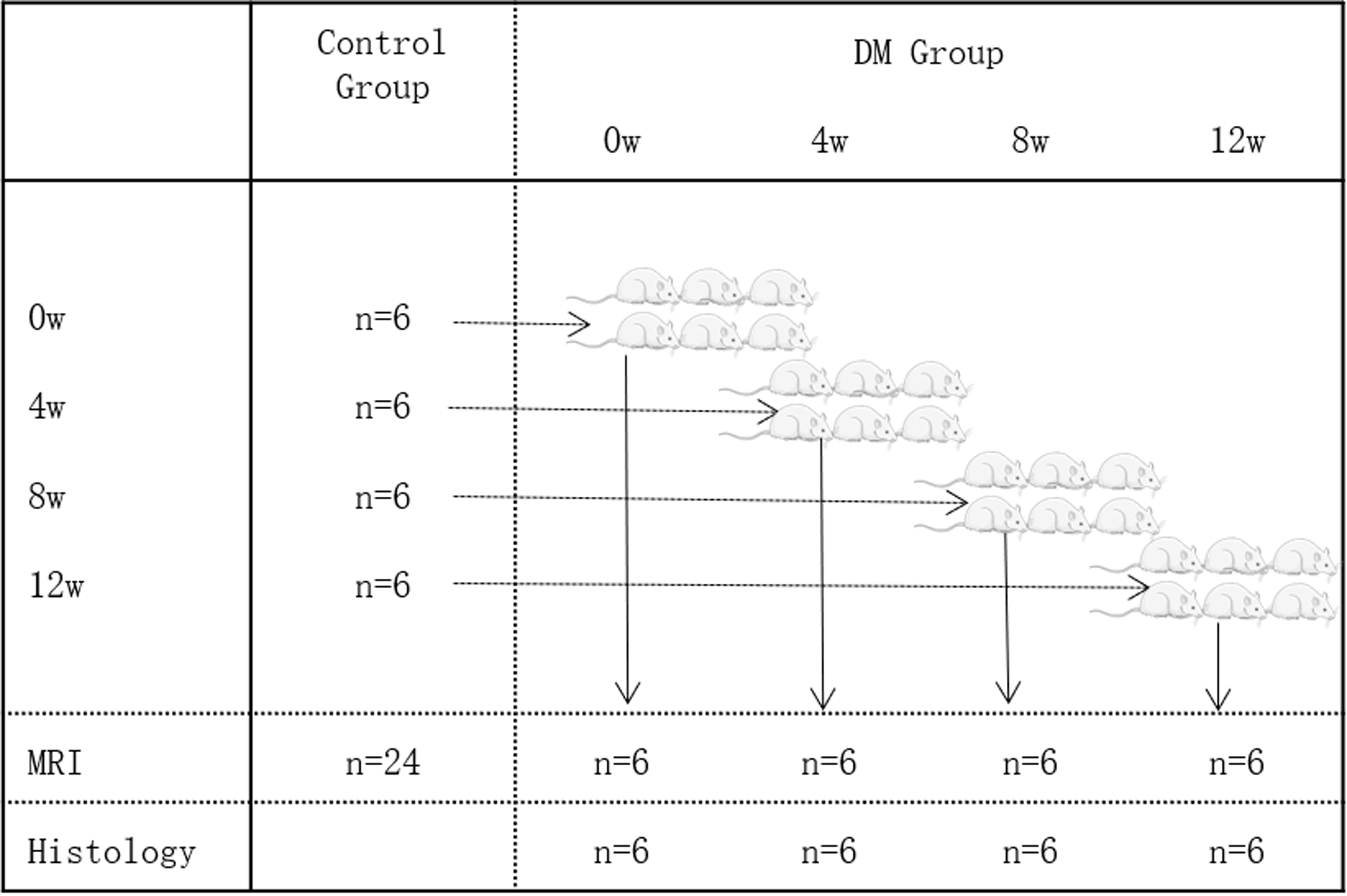

Supplement: Supplementary file 1 — Additional file 1: Supplemental Figure 1. Experimental protocol and allocations of rats to the two study groups. Six rats in each group underwent (1) streptozotocin or citrate buffer intraperitoneal injection, (2) Blood and urine tests, (3) MR scan (at 0, 4, 8, and 12 weeks after DM induction), (4) Blood and tests, followed by (5) histopathological examination. [file 12882_2021_2530_MOESM1_ESM.tif]
