# Supplementary material for: Non-invasive investigation of early kidney damage in streptozotocin-induced diabetic rats by intravoxel incoherent motion diffusion-weighted (IVIM) MRI
Source: BMC Nephrol. 2021 Sep 26;22:321. doi: 10.1186/s12882-021-02530-8 (PMC8474753; doi:10.1186/s12882-021-02530-8)
Supplement: Supplementary file 2 — Additional file 2: Supplemental Table. Expression of Collagen IV in renal tissue of the two groups of rats [file 12882_2021_2530_MOESM2_ESM.docx]

| **Supplemental Table Expression of Collagen IV in renal tissue of the two groups of rats** | | | | | | | | | |
| --- | --- | --- | --- | --- | --- | --- | --- | --- | --- |
|  | | Collagen Ⅳ expression | | | | | | |  |
| Group | | - | | 1+ | | 2+ | | 3+ | Total |
| 0w | control | 6 | 0 | | 0 | | 0 | | 6 |
|  | DN | 6 | 0 | | 0 | | 0 | | 6 |
| 4w | control | 6 | 0 | | 0 | | 0 | | 6 |
|  | DN | 0 | 4 | | 2 | | 0 | | 6 |
| 8w | control | 6 | 0 | | 0 | | 0 | | 6 |
|  | DN | 0 | 3 | | 3 | | 0 | | 6 |
| 12w | control | 5 | 1 | | 0 | | 0 | | 6 |
|  | DN | 0 | 0 | | 0 | | 6 | | 6 |
